# Comparative Efficacy of Second-Generation Antipsychotics vs. SSRIs in Managing Behavioral Symptoms in Children and Adolescents with Autism Spectrum Disorder

**DOI:** 10.1192/j.eurpsy.2025.1551

**Published:** 2025-08-26

**Authors:** H. M. Nguyen, L. Schmidt, B. Carr

**Affiliations:** 1 University of Florida College of Medicine, Gainesville, FL, United States; 2 Psychiatry, University of Florida College of Medicine, Gainesville, FL, United States

## Abstract

**Introduction:**

Autism Spectrum Disorder (ASD) is characterized by social interaction challenges, repetitive behaviors, and behavioral issues such as irritability, aggression, and hyperactivity. These symptoms lead to significant functional impairments, placing emotional and financial burdens on caregivers. Current treatment options include second-generation antipsychotics (SGAs) and selective serotonin reuptake inhibitors (SSRIs), but direct comparisons of their efficacy in managing ASD-related behaviors are limited.

**Objectives:**

This review aims to compare the efficacy and safety of SGAs and SSRIs in reducing behavioral symptoms in children and adolescents with ASD.

**Methods:**

A comprehensive systematic review by Lamy et al. (2020) evaluated over 50 studies on pharmacotherapy for ASD, including randomized clinical trials (RCTs) of SGAs like risperidone and aripiprazole. Other studies focused on specific SSRIs, such as citalopram, in ASD populations. Additionally, two notable trials were included: Ghanizadeh et al. (2014), which compared aripiprazole and risperidone, and King et al. (2009), a placebo-controlled study on citalopram.

**Results:**

Lamy et al. reported that SGAs, particularly risperidone and aripiprazole, significantly reduced irritability scores on the Aberrant Behavior Checklist (ABC) and Clinical Global Impression (CGI) scales (p<0.05), aligning with their FDA approval for ASD treatment. Ghanizadeh et al. (2014) also found that aripiprazole and risperidone reduced ABC scores (12.6 points for aripiprazole and 9 points for risperidone), though both were associated with side effects, such as increased appetite (34.5% for aripiprazole and 40% for risperidone) and drooling.

In contrast, King et al. (2009) found no significant improvement with citalopram over placebo (CGI-I improvement: 32.9% for citalopram vs. 34.2% for placebo) and noted more adverse effects in the SSRI group, including impulsiveness and insomnia. The review highlighted limitations, including methodological heterogeneity, lack of direct comparisons between SGAs and SSRIs, and variability in treatment duration.

**Table:**
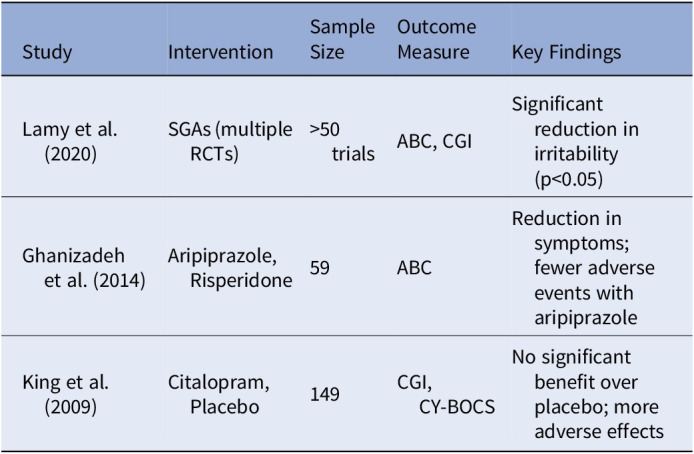

**Conclusions:**

In conclusion, SGAs appear more effective than SSRIs in managing ASD-related behavioral symptoms, particularly irritability. Despite limitations, SGAs show consistent benefits with a manageable safety profile. Future research should prioritize direct SGA vs. SSRI trials and longer treatment durations to inform clinical decision-making in ASD pharmacotherapy.

**Disclosure of Interest:**

None Declared

